# Biofilm Formation, *gel* and *esp* Gene Carriage among Recreational Beach Enterococci

**DOI:** 10.5539/gjhs.v6n5p241

**Published:** 2014-06-13

**Authors:** Ahmad Asmat, Ayokunle Christopher Dada, Usup Gires

**Affiliations:** 1 School of Biosciences & Biotechnology, Faculty of Science & Technology, Universiti Kebangsaan Malaysia, 43600 UKM Bangi, Selangor, Malaysia; 2 School of Environmental & Natural Resource Sciences, Faculty of Science & Technology, University Kebangsaan Malaysia, 43600 UKM Bangi, Selangor, Malaysia

**Keywords:** biofilm, *gel*, *esp*, beach water, beach sand, *enterococci*

## Abstract

Biofilm production, *gel* and *esp* gene carriage was enumerated among forty six vancomycin resistant *enterococci* (VRE) and vancomycin susceptible *enterococci* (VSE) beach isolates. A higher proportion (61.54%) of biofilm producers was observed among beach sand as compared to beach water *enterococci* isolates (30%) indicating that *enterococci* within the sand column may be more dependent on biofilm production for survival than their beach water counterparts. Correlation analysis revealed strongly negative correlation (r=-0.535, p=0.015) between vancomycin resistance and biofilm formation. Given the observation of high prevalence of biofilm production among beach sand and the concomitant absence of *esp* gene carriage in any of the isolate, *esp* gene carriage may not be necessary for the production of biofilms among beach sand isolates. On the whole beach sand and water isolates demonstrated clearly different prevalence levels of vancomycin resistance, biofilm formation, *esp* and *gel* gene carriage. Application of these differences may be found useful in beach microbial source tracking studies. Tested starved cells still produced biofilm albeit at lower efficiencies. Non-dividing *enterococci* in beach sand can survive extended periods of environmental hardship and can resume growth or biofilm production in appropriate conditions thus making them infectious agents with potential health risk to recreational beach users.

## 1. Introduction

Whether on nutrient-sufficient natural, industrial, or medical ecosystems, surface-associated bacterial growth are found everywhere (Costerton et al., 1995). However, despite their ubiquity, only recently did knowledge emerge on the genetics and physiology of these surface-associated bacteria (Kristich et al., 2004). Meanwhile, recent studies have clarified that surface-associated bacteria exist in complex microbial communities, known as biofilms, which are typically encased in a self-produced extracellular polymeric matrix (Stoodley et al., 2002). Typically, a biofilm is an assemblage of microbial cells that is irreversibly associated with a surface and enclosed in a matrix of primarily polysaccharide material (Donlan & Donlan, 2002). It is well documented that osmolarity, nutrient concentration and composition, among other environmental conditions influence the formation of surface-adherent biofilms by diverse bacterial species (Klausen et al., 2003; Loo et al., 2000; Qin et al., 2001; Rachid et al., 2000; Yoshida & Kuramitsu, 2002).

Biofilms can promote and sustain infections (Ramadhan & Hegedus, 2005). It was reported that biofilm bacteria are up to a 1,000 times more resistant to phagocytosis, antibodies and antibodies (Costerton et al., 1994). Among the associated explanations is the delayed penetration of antimicrobial agents through the exo-polysaccharide matrix, suppression of growth rate within the biofilm and production of a subpopulation of microorganisms in the biofilm that can develop into a phenotypic state that is highly protected (Stewart & Costerton, 2001). For example, biofilm forming *Enterococcus faecalis* have been shown to survive better in macrophages than negative isolates (Lucilla et al., 2004). In a previous study, the capacity to form biofilms was found to be common among clinical *E. faecalis* isolates particularly within the subpopulation carrying the *esp* gene which is believed to promote primary attachment of and biofilm formation by *E. faecalis* on abiotic surfaces (Toledo-Arana et al., 2001). On the other hand, however, Dworniczek et al. (2003) and Mohamed et al. (2004) reported in their studies that the *esp* gene was not required for biofilm formation. In addition to the presence of *esp*, another recent study presented data that supports the hypothesis of in vitro biofilm production by *E. faecalis* in the absence of the whole pathogenicity island harbouring the *esp* coding sequence (Kristich et al., 2004).

A number of studies have explored the formation of biofilm among enterococci and its relation to the presence of one or more putative factors (Toledo-Arana et al., 2001; Kristich et al., 2004; Rosa et al., 2006; Raad et al., 2005; Mohamed et al., 2004; Tendolkar et al., 2004; Leavis et al., 2004; Heiken et al., 2007; Paganelli et al., 2013; de Paz et al., 2011). Most of these isolates were recovered from clinical laboratories. There is a scarcity of published information on biofilm formation among enterococci recovered from beach water and sand isolates. Agreeably, the many available studies reported in literature, generally in clinical setting is justifiable given that biofilms have been implicated as etiological agents of chronic infection (Costerton et al., 1999; Donlan & Costertan, 2002). However, in environmental settings, the case is less clear. However, given the blurred lines of demarcation between clinical and community settings, particularly as it relates to waste management in developing nations (Dada et al., 2013), there may be the possibility of potentially virulent environmental enterococci being ignored but yet existing in hitherto unconsidered environments. Such is beach water available for recreational purposes. In the light of the backdrop of relatively fewer studies on *Enterococci* recoverable from beach water or beach sand that are used for recreational studies, there is a general dearth of information available on biofilm production among environmental *Enterococci*. Our study set out (i) to determine the ability of vancomycin susceptible *enterococci* (VSE) and vancomycin resistant *enterococci* (VSE) from recreational beach water and sand to form biofilms, and (ii) to show any correlation between biofilm formation and the presence of the *gel* and *esp* genes.

## 2. Methods

### 2.1 Enumeration of Enterococci Isolates

Isolates were part of those recovered during a national surveillance study on recreational beaches. A total of forty-six (46) colonies consisting of 20 beach water and 26 beach sand *enterococci* isolates were collected for the study. These were purified by further sub-culturing on Slanetz and Bartley (S + B) and on Brain-Heart Infusion agar. Preliminary confirmation was done to confirm the genus *Enterococcus* by checking that isolates taken from Brain– Heart Infusion agar hydrolyse bile esculin, grow in 6.5% NaCl and in BHI at 45 °C. As described by previous reports (Facklam, 2002; Facklam & Elliott, 1995), selected biochemical tests were conducted on the selected isolates. These included carbohydrate fermentation with 1% mannitol, sorbitol, arabinose, raffinose, sucrose, lactose and inulin. Motility Test Medium was also done using SIM agar (Oxoid, UK).

*Enterococci* isolates selected for this study were confirmed using the *rpoA* gene (Naser et al., 2005). Using a modified version of Creti et al. (2004), enterococcal DNA was prepared by introducing a loop of colonies from BHI agar plate into 200 µl of sterile distilled water in pcr tubes. A boiling protocol of 95 °C for 15 mins was applied after which tube contents were briefly centrifuged at 13,000 rpm for 1min, longer times of centrifugation was observed to denature the DNA in the study. Tubes were transferred into ice and subsequently, the supernatant containing the genome was removed and dispensed into new sterile tubes. An aliquot of 5 µl of the recovered supernatant was used in a final volume of 50 µl of pcr mixture. Each 50 µl pcr mixture used contained 1X PCR buffer, 2 mM MgCl, 200 µm each DNTP, 0.25 U of YEAtaq DNA Polymerase and 0.2 µM of *RpoA* primers (forward 5’-ATGATYGARTTTGAAAAACC-3’ and reverse 5’- ACHGTRTTRATDCCDGCRCG-3’). Amplification was done using DNA thermal cycler (Eppendorf AG, Hamburg, Germany) consisting of an initial activation step at 95 °C for 4mins followed by 30 cycles of denaturation at 94 °C for 1min, annealing at °C for 1min, extension at 72 °C for 1min and a single cycle of 7 mins at 72 °C.

Subsequently, 10 µl of pcr product obtained were mixed with 2 µl of orange gel loading buffer and electrophoresed to detect the presence of the 112 bp pcr product. A 100bp DNA ladder (Fermentas) was also included as molecular size marker in the electrophoresis *gel* run). PCR amplicons of tuf genes from the recovered strains were confirmed by DNA sequencing with an ABI 3130XL 20 genetic analyzer (Applied Biosystems). PCR amplicons were purified using QIAquick PCR Purification Kit (Qiagen). Purified amplicons of *rpoA* sequences were blasted for sequence similarity to annotated sequences at http://www.ncbi.nlm.nih.gov. The phylogenetic relationships among species were analyzed using the neighbor-joining method in MEGA 5.0 (Tamura et al., 2011). Distances between the sequences were calculated using Kimura’s two-parameter model. Levels of similarity were determined among species. Bootstrap values were obtained for 1000 randomly generated trees.

### 2.2 Biofilm Formation

Two approaches were used. First, the qualitative method to detect biofilm production using Congo Red Agar (CRA) medium as reported by Mathur et al. (2006) was adopted. CRA medium was prepared with brain heart infusion agar supplemented with congo red (8 g/L) and 3.6% (w/v) saccharose. CRA plates were inoculated with test organisms and incubated at 35 °C for 24 h aerobically. Black colonies with a dry crystalline consistency indicated biofilm production (Reid, 1999). Second, the quantitative ability of the *Enterococci* isolates to form a biofilm on an abiotic surface was quantified as described by Elhadidy and Elsayyadas (2013). After growth overnight in brain heart infusion broth (BHIB) at 35 °C, 200 μl of the suspension already diluted to 1:40 was introduced in triplicates into sterile 96-well polystyrene microtiter plates (Sigmaaldrich, USA). Following a 24 hours period of incubation at 37 °C and washing of wells with 200 μl phosphate-buffered saline, the wells were dried in an inverted position for 1 hour. Subsequently, staining with 1% crystal violet (CV) was done for 15 minutes at room temperature. After washing off excess stain, CV was extracted from adhering bacterial cells using 200 μl of 80:20 (v/v) ethyl alcohol/acetone. This was followed by OD reading at 570 nm (OD_570_) using microplate ELISA reader (BioRad, USA). Obtained OD readings were converted to binary coding system based on a criteria defining 0 as equals to non-biofilm formation (OD_570_ is <0.5) and 1 as equals to biofilm formation (OD_570_ is >0.5). Isolates that yielded scores of one in both congo red and crystal violet assays were considered as true positives for biofilm production. To detect the effect of starvation on biofilm production, isolates producing biofilm were subjected to a period of cultivation in 1ml of BHIB for 3weeks at 35 °C. Starved isolates were subsequently tested for biofilm production.

### 2.3 Detection of esp and Gel Genes

All strains of *enterococci* included in the study were screened for the presence of *gel* and *esp* gene using polymerase chain reaction (PCR). An aliquot of 5 µl of the template DNA from cell lysis methodology previously described was used in a final volume of 50 µl of pcr mixture. Each 50 µl pcr mixture used contained 1X PCR buffer, 2 mM MgCl, 200 um each DNTP and 0.25 U of YEAtaq DNA Polymerase. *Gel* (forward 5’-TATGACAATGCTTTTTGGGAT-3’, reverse 5’-AGATGCACCCGAAATAATATA-3’ and *Esp* (forward 5’-AGATTTCATCTTTGATTCTTGG-3’, reverse 5’-AATTGATTCTTTAGCATCTGG-3’) primers were synthesized by First Base Sdn. Bhd. and reconstituted to final concentrations of 0.1 µM and 0.2 µM respectively. For each primer, initial optimization experiments were conducted to ascertain optimal pcr conditions for MgCl and annealing temperatures. Amplification was done using DNA thermal cycler (Eppendorf AG, Hamburg, Germany) consisting of an initial activation step at 95 °C for 4mins followed by 30 cycles of denaturation at 94 °C for 1 min, annealing at 56 °C for 1 min, extension at 72 °C for 1min and a single cycle of 7 mins at 72 °C. Subsequently, 10 µl of pcr product obtained were mixed with 2 µl of orange gel loading buffer and electrophoresed to detect the presence of the 213 bp and 510 bp pcr product respectively for *gel* and *esp* genes. A 100 bp DNA ladder (Fermentas) was also included as molecular size marker in the gel. Reference strain (*E. faecalis* MMH594) was gratefully provided by Dr Fatima Lopez (Instituto de Tecnologia Química e Biológica, Universidade Nova de Lisboa, Portugal). Purified PCR amplicons of *esp* and *gel* genes from the recovered strains were confirmed by DNA sequencing with an ABI 3130XL 20 genetic analyzer (Applied Biosystems). The DNA sequences were blasted for sequence similarity to annotated sequences at http://www.ncbi.nlm.nih.gov.

### 2.4 Detection of Vancomycin Resistance among Enterococci

Vancomycin resistance among encountered *Enterococci* isolated was determined in line with the National Committee for Clinical Laboratory Standards by agar dilution method using VRE agar base supplemented with 6 g/L vancomycin. Black colonies of isolates that grew in the presence of the antibiotic supplement were considered as resistant while others were considered as susceptible.

### 2.5 Statistical Analysis

Taking each of the considered parameters as categorical variables, Fisher’s exact test was used to determine if there are non-random associations between any two categorical variables. All results from the tests conducted were converted to a binary coding system using ‘0’ for sensitive and intermediate or negative as in the case of absence of biofilm formation or lack of carriage of *esp* or *gel* genes, whereas ‘1’ represented vancomycin resistant phenotypes or positive as in the case of biofilm formation or carriage of *esp* or *gel* genes. Obtained phenotypic and genotypic profile for each isolates were analysed using unsupervised, average-linkage, hierarchical clustering (PAST) employing Pearson correlation coefficient and binary squared Euclidean distance measure to determine relatedness of each isolate. The closest isolates were merged in an agglomerative way by identifying the pairs of cases that were most similar to each other, as determined by their correlation coefficient across all assays conducted. Clusters were observed from plots of dendogram produced.

### 2.6 Nucleotide Sequence Accession Numbers

The sequences obtained in this study have been deposited in GenBank and have been assigned accession numbers: beach water isolates (*rpoA* gene: KC963146, KC963147, KC963148, KC963149, KC963150, KC963151, KC963152) and beach soil isolates (*rpoA* gene: KC963138, KC963139, KC963140, KC963141, KC963142, KC963143, KC963144, KC963145).

## 3. Results

**Figure 1:**
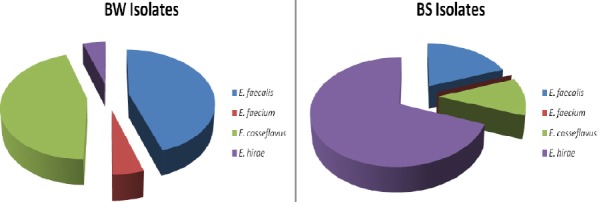
Species differentiation of beach water and beach sand isolates tested in this study

**Figure 2:**
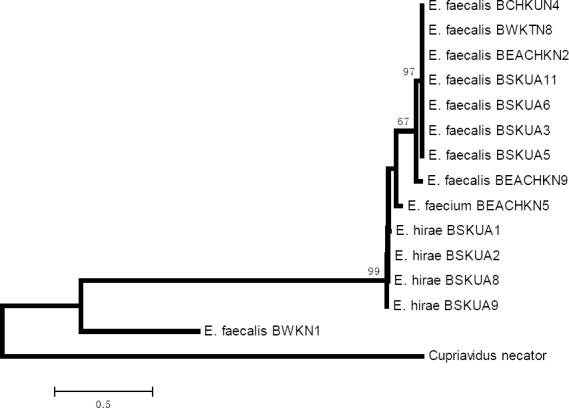
Phylogenetic relationship between the concatenated sequences of *rpoA* gene obtained for beach water and beach sand *enterococci* isolates

**Figure 3:**
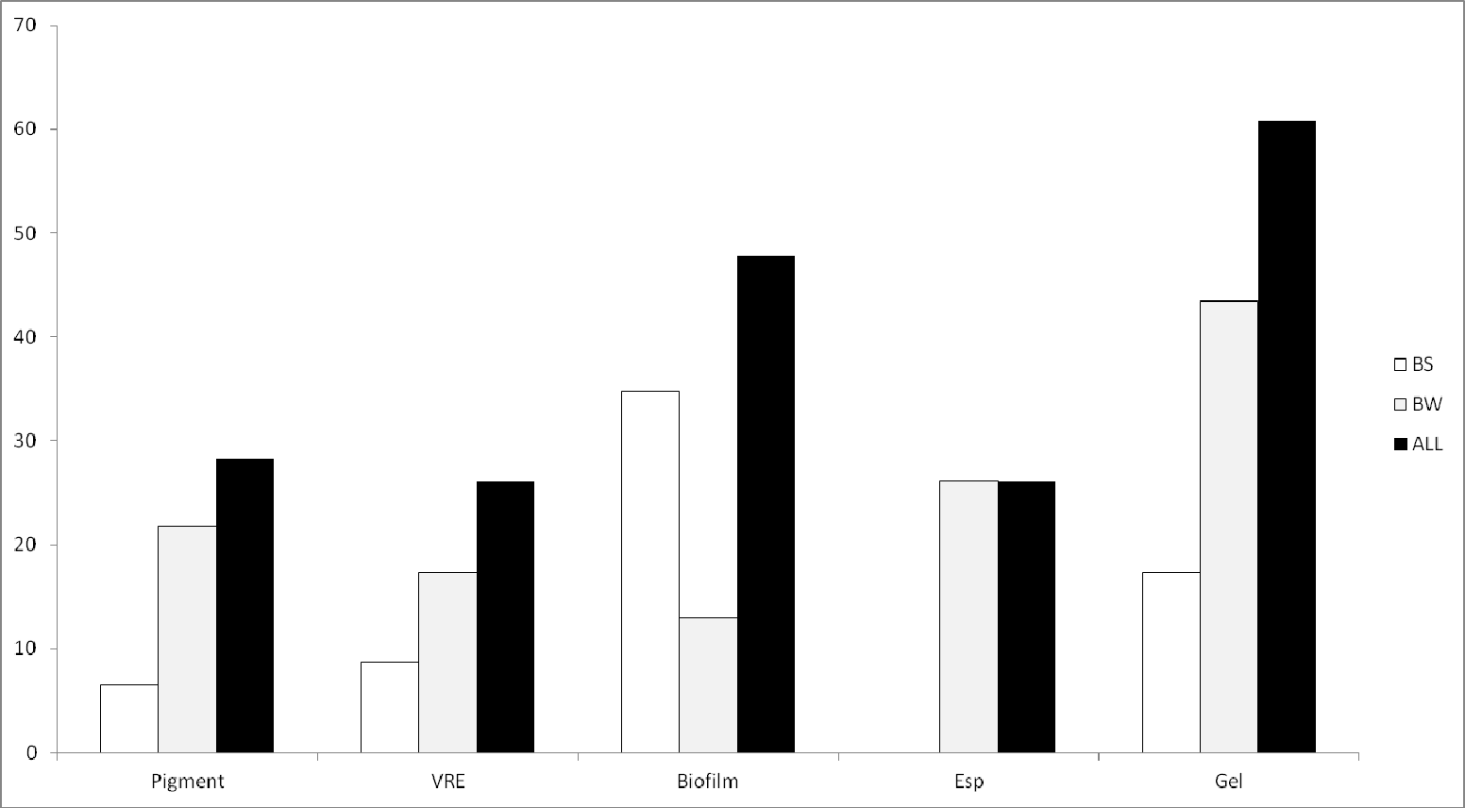
Proportion of beach water and beach sand *Enterococci* observed positive for each of the factors tested

In this study, biofilm production among a population of vancomycin resistant (VRE) and susceptible (VSE) *enterococci* was investigated among beach sand and beach water isolates. Isolates of presumptive *enterococci* based on biochemical tests were confirmed using *rpoA* sequencing. In terms of the speciation of *Enterococci* from the randomly selected population of species tested, the frequency of occurrence for E*. faecalis* and *E. casseflavus* was highest (both 45%) while the least was *E. faecium* and *E. hirae* (Figure 1). No other species was detected apart from these four species among the beach water (BW) isolates. On the contrary, in the case of beach sand (BS), the proportion of *E. hirae* (69.23%) was the highest followed by *E. faecalis* (19.23%). The proportion of *E. casseflavus* recovered among BS isolates was least. Results obtained from *rpoA* gene sequencing were in agreement with those of the biochemical tests. Using a neighbour joining approach that incorporates 1000 bootstrap replications, a phylogenetic tree based on *rpoA* partial sequences of *Enterococci* isolates recovered from recreational beach water and sand is shown in Figure 3. *RpoA* sequence analysis showed good distinctions for intra-species variations among *E. faecalis* (Figure 2). Two major *rpoA* allele types among *E. faecalis* were observable from the *rpoA* sequence analysis.

On the whole, the proportion of *Enterococci* possessing virulence properties from both sources was highest based on the presence of gelatinase gene (60.87%) and least based on the presence of *esp* gene (26.09%) (Figure 3). In terms of isolate source, a higher proportion of BW isolates were vancomycin resistant and carried *gel* gene as compared to their BS counterparts. On the other hand, a higher proportion of BS isolates were biofilm producers.

**Figure 4:**
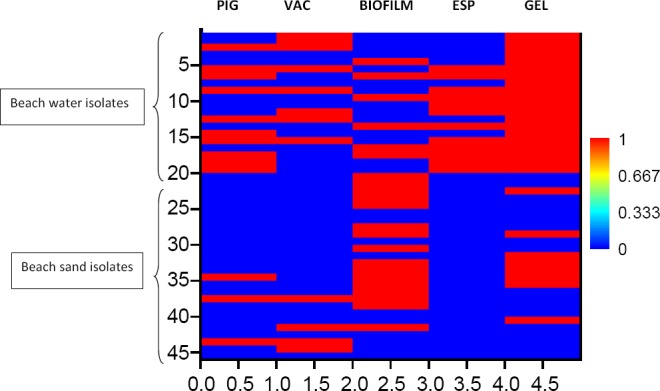
Heat map showing a pictorial reparation of the binary matrix obtained for beach water and beach sand *Enterococci* after each factor testing

Using a colour gradient that ranged from 0 (blue) to 1(red) for negative or positive response respectively for assays for each categorical variable, the response heat map of the isolates considered in this study is presented in Figure 4. From the map, isolates from beach water were coded in such a way that they ranged from points 0 to 20 on the y-axis while BS isolates ranged from point 21-46. BW isolates comprised generally of equal proportions of pigmented and non-pigmented *Enterococci*. Also, most isolates recovered from beach water carried the *gel* gene. This was observably different for beach sand isolates where a higher proportion of non-pigmented isolates carried the *gel* gene than was observed among pigmented isolates.

In terms of vancomycin susceptibility and biofilm formation among beach water isolate, the heat map did not present any identifiable pattern. A reliable conclusion was thus made based on the correlation analysis. Pearson Correlation analysis revealed strongly negative correlation (r=-0.535, p=0.015) between vancomycin resistance and biofilm formation among beach water isolates. Meanwhile, among the beach soil isolates (y=20 to 46), absence of vancomycin resistance was in most cases linked to the production of biofilm based on the colour pattern obtained.

A higher proportion (61.54%) of biofilm producers was observed among beach sand as compared to beach water enterococci isolaltes (30%). *Enterococci* within the sand column thus seem to be more dependent on biofilm production for survival than their beach water counterparts. Eight of the 20 tested beach isolates were found to be resistant to vancomycin while 5 of the tested 26 isolates of beach sand were resistant to vancomycin. Correlation analysis revealed strongly negative correlation (r=-0.535, p=0.015) between vancomycin resistance and biofilm formation. While none of the each water isolates that produced biofilms were simultaneously resistant to vancomycin, two out of the five encountered VRE among beach sand isolate produced biofilm. All beach water isolates possessed *gel* gene while only 40% of their beach sand counterparts possessed the gene. On the other hand, a high proportion of isolates that carried the *esp* gene was observed among beach water isolates while the presence of this gene was not detected among beach sand isolates. Coupled with the observation that high prevalence of biofilm production among beach sand and the concomitant absence of *esp* gene carriage in any of the isolate, *esp* gene carriage may not be necessary for the production of biofilms among beach sand isolates. On the other hand, five out of the six isolates beach water isolates that produced biofilm possessed *esp* gene suggesting that this gene may be necessary for the production of biofilms among beach water enterococci. On the whole beach sand and beach water isolates tested demonstrated clearly different prevalence of vancomycin resistance, biofilm formation, *esp* and *gel* gene carriage.

**Figure 5:**
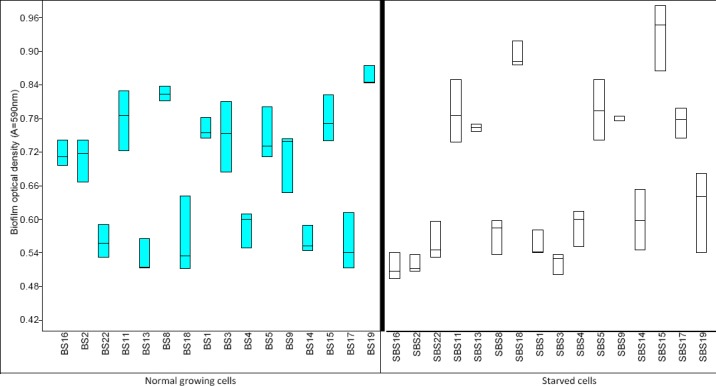
Comparative biofilm formation among beach soil (BS) isolates growing normally and beach soil isolates starved for a period of 3 weeks (SBS)

**Figure 6:**
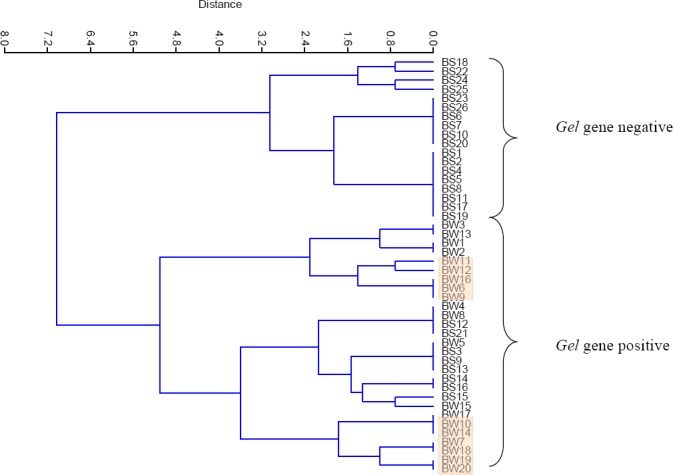
Hierarchical clustering showing two major clusters among the tested beach water and beach sand isolates. Shaded potions indicate *esp* gene carrying *enterococci*

The effect of starvation among tested enterococci isolates was observed among the biofilm producing beach soil isolates. Starved cells of beach soil still produced biofilm. However for nine out of 16 tested isolates, biofilm produced had optical densities lower than their unstarved counterparts (Figure 5). An attempt was made to adopt a multivariate approach to cluster the patterns observed among the tested isolates. The obtained dendogram grouped all *gel* positive and all *gel* negative isolates into two separate clusters (Figure 6). Using a cut-off point of 50% of the total distance obtainable in the tree, it was observed that *gel* gene carriage was more prevalent among *Enterococci* recovered from beach water. The *esp* positive *Enterococci* tested in this study formed sub-clusters among the main cluster of *gel* positive *Enterococci*.

## 4. Discussion

Testing bathing beach water for microbial contamination is necessary to ensure that public health is not put at risk from pathogens. EPA recommends the use of *enterococci* as the basis for water quality criteria for bacteria (EPA 2003, 2011). *Enterococci* are members of the genus *Enterococcus* (Moellering, 1992). They are spherical, gram-positive and grow in chains. Most members of the group are facultative, “aerotolerant” anaerobes. *Enterococci* are normal flora of the gastrointestinal tract, where they are present at high concentrations. However, apart from being indicator organisms, they can cause opportunistic infections in immune-compromised individuals when they leave the gastrointestinal tract. Notable among such are bacteremia, wound infections, urinary tract infections and endocarditis (Dada et al., 2013). Recently, *Enterococci* have been implicated in nosocomial infections. Many nosocomial infections result from the ability of microorganisms to form biofilms. The molecular mechanisms involved in enterococcal biofilm formation have not been fully explored and are only now beginning to be understood (Tendolkar et al., 2004). We investigated in this study the ability of vancomycin susceptible *enterococci* (VSE) and vancomycin resistant *enterococci* (VSE) from recreational beach water and sand to form biofilms, and any correlation between biofilm formation and the presence of the *esp* and *gel* gene.

In this study, we identified using a combination of biochemical tests, four major species of *Enterococci*. A previous study however opined that biochemical tests do not suffice in the identification of *Enterococci* (Knudtson et al., 1992). These inherent inadequacies arise because not all strains within a given species may exhibit a common characteristic, (ii) the same strain may give different results upon repeated testing, (iii) small alterations in the execution of an assay may give false test results (Bosshard et al., 2004). This explains why further confirmation was done on at least 30% of the isolates considered in the study. Obtained results from *rpoA* sequence analysis correlated with the results obtained using biochemical tests. The observed correlation of biochemical tests for speciation of *Enterococci* with results obtained by genotyping in this study may however carry some potential for bias considering the size of our sample population. For a larger library of isolates, chances exist of encountering a more diverse species of *Enterococci* which may present considerable difficulties for the adoption of a phenotypic approach in speciation.

Nonetheless, we noticed that while *E. faecalis* and *E. casseliflavus* dominated beach water isolates, *E. hirae* dominated beach sand isolates. In similar study conducted a tropical location, the dominant *Enterococci* were identified as *E. casseliflavus* in both the river and the beach samples regardless of the season or location suggesting their adaptation to this environment when compared to the other *Enterococci*. In the same study, among the non-pigmented, *E. faecalis w*as the dominant *Enterococci* (Cuevas-Irizarry, 2011). This observation is similar to our findings. Preliminary information on the beach where samples were collected for *Enterococci* isolation revealed the presence of a zoo near to the beach. This probably explains the predominance of *E. hirae* in beach sand isolates.

Considering the response obtained for each of the tested variables in this study, the highest proportion was observed to be *gel* gene carriage. It could thus be inferred that this factor may be fairly universally distributed among environmental *enterococci*. However, the expression of this gene among environmental isolates appear to be silent as none of the *gel* positive isolates were able to hydrolyse gelatinase in gelatinase-supplemented BHI agar in this study. A major weakness of this study was the non-inclusion of clinical data in the analysis to confirm the validity of this observation among non-environmental *enterococci* isolates. Also, we did not check for the presence of the quorum-sensing system encoded by the fsr gene cluster as reported by Nakayama et al. (2002). Nakayama et al. (2002) reported a strong association between a 23.9-kb chromosomal deletion containing the fsr gene cluster region and prevalence of gelatinase-negative clinical isolates among *E. faecalis* from urine. Nonetheless, the widespread presence of gelatinase among isolates regardless of their vancomycin susceptibility or biofilm production or *esp* gene carriage questions the appropriateness of the inclusion of the gene as a virulence marker among *Enterococci*. In this direction, Gulhan et al. (2006) opines that although important virulence factors such as *gelE* may mediate the hydrolysis of gelatine, casein, haemoglobin, and other bioactive peptides, *gelE* may not be considered an important virulence factor by itself. The higher prevalence of gelatinase positive Enterococci among BS isolates as compared to BW isolates in this study seem to suggest the carriage of gelatinase gene as more as an adaptive factor enhancing the ability to breakdown organic-rich nutrients in beach sand.

In our study, a higher proportion of beach water isolates than was observed among beach soil isolates were vancomycin resistant. Additionally, the presence of vancomycin resistance (observable among beach water isolates) was almost always linked to the absence of biofilm production which was prevalent among beach soil isolates. Thus it is suggestively opined that isolates (i) lose their biofilm production ability as they take up vancomycin resistance and (ii) *Enterococci* within the sand column seem to be more dependent on biofilm production for survival than their beach water counterparts. The matrixes in beach sand present a favourable mix of organic nutrients. Yet beach soil endures major cycles of caking or drying given the intense daily temperatures (often reaching up to 35 °C) and wetting and dislodgement by tidal waves from the adjacent beach water. Arguably, sand enterococci thus have to develop better adaptation measures to resist drying or washing off by ocean waves. According to a report by the WHO (2009), the survival of enteric bacteria on the surface of dry sand is suggestively however of short duration, the bacteria being destroyed mostly by environmental pressure. A possible explanation could be that those that survive are better adapted to surviving tougher environmental pressure including antibiotics and dispersal mechanisms.

Additionally, constant turbulence caused by bathers during recreational activities are responsible for most of the dispersal and deviation from equilibrium of *Enterococci* population within beach soil column. A number of studies have attempted tracing movements of sediments in the coastal zone. In a pioneering study by Trask (1952) on the investigation of the movement of beach sand along the southern California coast, it was demonstrated that sand at the considered beach may have come from a distance of more than 160 km up the coast. More recently, variations in the degree of grain rounding have been used to trace sand movements, or to obtain additional information concerning the history of the sediment particles (Allan & Hart, 2007; Komar, 1992, Asmat et al., 2014). The findings of these studies however present a number of questions concerning sources of beach sands and fate of microbial particles. Having highlighted this, the markedly differing characteristics observed among beach water and beach sand isolates in this study may be useful for application in microbial source tracking studies that aim to decipher if faecal enterococci loadings in adjacent waters are attributable to beach soil. Other striking questions this study raises for future research are: is biofilm production an adaptive strategy among *enterococci* recoverable from beach sand? Could it be that this factor helps the organisms in coping with the effect of dislodgement from nutrient sources that may be associated with dispersal of beach sand? Hopefully more studies will emerge in the future that will attempt to answer this question using a large pool of beach sand *Enterococci* exposed to varying levels of dispersal and environmental pressure.

All *enterococci* isolates were also screened for the presence of *esp* gene. Ahmed et al. (2007) tested 197 faecal samples from 13 host groups in Southeast Queensland, Australia and concluded that the *esp* marker appears to be sewage specific and could be used as a reliable marker to detect human faecal pollution in surface waters. The presence of *esp* gene was least among the proportions observed for all isolates in this study. In other words, it was not commonly encountered among beach *enterococci* in the current study. These observations, along with previous studies that have documented association of the presence of this gene with isolates from faecal sources indicate the specificity of this gene. In our study, *esp* gene was not detected among BS isolates. A major observation in terms of *esp* gene carriage in our study thus presents implication in terms of faecal pollution inputs into seawater available for recreational purposes. While arguably, a school of thought opines that beach sand may be the cause of elevated *enterococci* loadings in adjacent seawater, the case here is somewhat different. *esp* is normally related to human faeces (Scott et al., 2005), yet it was observed present in most of the beach water isolates and not among beach sand isolates. A possible explanation for this is that faecal *enterococci* loading into the considered beach water may be from other sources excluding beach sand. This could include hidden sewage pipes that supply *esp* positive, faecal *enterococci* into the seawater. Interestingly, among the beach water isolates where *esp* was detected, biofilm production was associated with the possession of the *esp* gene unlike beach soil isolates where biofilm production was independent of the possession of the *esp* gene. It may thus be inferred that *esp* gene carriage may not be necessary neither is it sufficient for the production of biofilms among the beach soil *Enterococci* while the opposite is true for beach water isolates.

Considering results from the biofilm producing beach sand isolates exposed to different physiological states, our study showed that starved enterococci cells are still able to form biofilms but with reduced efficiency as compared to growing cells. This observation is in concert with the findings of Lleo et al. (2007). All starved cells in our study were recoverable on BHI agar as viable cells after the period of 3 weeks starvation in BHIB. However, during the period of starvation, Enterococci cells settled down the bottom of the tube as a precipitated mass. During this stage, beach sand enterococci were able to survive albeit without active division unlike their growing cell counterparts that were tested during the exponential growing phase. Our results thus indicate the possibility of Enterococci in beach sand surviving extended periods of environmental hardship such as nutrient starvation. These non-dividing bacterial forms may subsequently play roles as infectious agents thus constituting a risk to human health.
